# A Rose by any Other Name? Using Core Components to Categorize Social and Emotional Learning Provision

**DOI:** 10.1007/s12310-023-09585-y

**Published:** 2023-05-11

**Authors:** Michael Wigelsworth, Carla Mason, Lily Verity, Neil Humphrey, Pamela Qualter

**Affiliations:** Manchester Institute of Education, University of Manchester, Manchester, M13 9PL England

**Keywords:** Social and emotional learning, SEL, Core components, Universal intervention, Common elements

## Abstract

Although social and emotional learning (SEL) benefits children and youth worldwide, classifying a program as SEL is insufficient to capture its variability of content. There is currently little to aid in identifying specific program content so that foci may be identified (e.g., self-management skills vs. social skills). This gap poses a difficulty for researchers attempting to address heterogeneity in SEL research and practitioners who want to select programs best suited for their contexts. This paper begins to address these concerns by extracting and contrasting ‘core components’ of interventions within an identified shortlist of 13 universal, elementary evidence-based programs through a distillation method using the often cited ‘five core competency’ model from CASEL (Collaborative for Academic, Social, and Emotional Learning). Results showed that CASEL’s core competencies are represented across short-listed programs. However, almost all programs had identifiable foci, targeting a subset of skills. Accordingly, the use of ‘core components’ is recommended as a method for offering more nuance in SEL classification for programs beyond the current study, with implications for program implementation and the design of future research in SEL evaluation.

## Introduction

Social and emotional learning (SEL) is the process whereby children and young people learn a broad range of social, emotional, and behavioral skills (Durlak et al., 2015). SEL is arguably a global phenomenon (Brush et al., 2022). Robust evidence supports its benefits for many outcomes, including immediate positive impacts on pupil well-being, behavior, and attainment (Osher et al., 2016), alongside socio-economic benefits later in life (Bellfeld et al., 2015). SEL encapsulates diverse intra- and inter-competencies (e.g., emotional self-management, social skills, empathy, and responsible decision-making, as discussed further below). However, despite this manifold approach, there is arguably a lack of nuance in presenting these skills in evaluation evidence. For instance, those wishing to implement SEL practices in school settings are directed to summative pools of evidence, noting which programs have robust historical success, but do little to indicate the specific competencies or skills addressed within individual program content (see, for example, CASEL-select’s catelogue (https://pg.casel.org). Therefore, there is a potential risk that implementers may assume that SEL programs typically address all competencies. Even without assuming an SEL program offers content in all key areas. Practitioners are still not optimally equipped to select the best program for their contexts without further information.

Furthermore, although the current field has a good understanding of *which* programs are effective, there is comparatively little understanding regarding precisely *what* skills programs address. This lack of scope is particularly pertinent in the context of SEL evaluation, given the long-standing concern about addressing heterogeneity in effectiveness (Corcoran et al., 2018; Durlak et al., 2011; Wigelsworth et al., 2016). SEL meta-analyses have noted an overall positive impact but with a high degree of variation, attributable to differences in the focus of individual programs (e.g.,Corcoran et al., 2018; Durlak et al., 2011; Sklad et al., 2012; Wigelsworth et al., 2016). Greater nuance in SEL program classification would help address a lack of detail presented to those researching or practicing SEL by establishing different foci in SEL programs for further consideration.

As a method to facilitate addressing these concerns, there is an opportunity to identify core components within programs, examine the extent to which particular topics, pedagogical approaches, or both are emphasized, and, in doing so, examine whether commonalities or differences occur across interventions. Early work in the field (e.g., Jones et al., 2017; Lawson et al., 2019; Wigelsworth et al., 2022) is promising, but there has not been a direct and explicit comparison of respective program content using CASEL’s core competencies.

The current study addresses this shortcoming by exploring the content within 13 evidence-based SEL programs. Thus, programs can be categorized beyond the singular classification of SEL relative to their core components.

### Social and Emotional Learning (SEL) as an Umbrella Term

‘Social and emotional learning’ has served as an umbrella term for diverse approaches that intervene across skills and competencies broadly related to intrapersonal management (e.g., emotional & behavioral regulation) and interpersonal skills (e.g. pro-social behavior & anti-bullying; Osher et al., 2016). A dominant approach in defining SEL is through five core (and inter-related) competencies: (1) self-awareness, (2) self-management, (3) social awareness, (4) relationship skills, and (5) responsible decision-making (Collaborative for Academic, Social and Emotional Learning [CASEL], 2002) and their corresponding specific skills (see Table 1). Although CASEL is not the only framework for organizing personal and social skills relating to social-emotional competence (see, for example, Schoon, 2021), it is arguably the most ubiquitous and, critically, is directly related to the delivery of evidence-based programmatic elements of SEL. Hence, CASEL’s adoption for the current study. Further, each of CASEL’s five core competencies and related skills fit well within current models of emotional understanding (see Castro et al., 2016). On a more practical note, CASEL publishes program evidence guides of SEL programs, noting histories and comparing the robustness of evaluations, similar to other ‘what works’ databases (e.g., https://www.blueprintsprograms.org/). Such an approach is consistent with a contemporary paradigm in respect to adopting evidence-based practice (Slavin, 2002).

**Table 1:** CASEL’s Core competencies and associated specific skills

Broad construct	Core competency	Specific skills
Intra-personal skills	Self-awareness (SA)	Identifying emotions; Accurate self-perception; Recognizing strengths; Self-confidence; Self-efficacy
	Self-management (SM)	Impulse control; Stress management; Self-discipline; Self-motivation; Goal setting; Organizational skills
Inter-personal skills	Social awareness (SOC)	Perspective taking; Empathy/sympathy; Appreciating diversity; Respect for others
	Relationship skills (RS)	Communication; Social engagement; Relationship building; Teamwork
Responsible decision making	Responsible decision making (RDM)	Identifying problems; Analyzing solutions; Solving problems; Evaluating; Reflecting; Ethical responsibility

A common feature among evidence-based SEL interventions includes the universality of the intended population and the broad modality of delivery. For instance, as a preventative skill-based approach (Elias & Weissberg, 2009), SEL is typically delivered universally to all children. Delivery modes are commonly through classroom-based practices and explicit teaching, which typically takes the form of a ‘teachers manual’ with accompanying stimulus materials for class use. For instance, in CASEL’s program guide for effective Social and Emotional Learning Programs, 15 of the 23 recommended programs contain taught curricula, and every program has a classroom component (e.g., teachers’ modeling skills). However, given the broad scope of SEL’s definition, there is significant content variation within SEL-based interventions. Some interventions focus on a particular set of skills (e.g., relationship building and teamwork), whereas others offer broader curricula across multiple competencies. Therefore, the current approach to SEL classification and evaluation possesses some difficulties.

### Need for a More Nuanced Approach to Describing SEL Programs

Through a single umbrella term to collate different interventions and an emphasis on summative and aggregative data for evidence of success, there is an assumed generalization by which SEL programs are sufficiently similar to be united under a single title. This ‘one size fits all’ approach may be true to a certain extent, by which commonality of program provision offers universally beneficial assets and knowledge. However, such an expansive approach is arguably problematic, inaccurate, inefficient, and incomplete.

It is inaccurate to take a summative/reductionist approach to SEL provision. Although there is a general expectation of broad coverage of integrated competencies, given their size and complexity, programs often comprise of a number of different foci, including different emphases regarding core competencies. For instance, ‘KiVa’ (Salmivalli et al., 2009) has the main aim of reducing bullying through a focus on social skills, whereas FRIENDS (Barrett, 2005), identified as an SEL intervention (Kozina, 2021), explicitly focuses on emotional regulation. Further nuance is arguably required to judge expected outcomes for such programs (for instance, FRIENDS would compare poorly to other SEL programs if social skills were assessed vs. emotional regulation). Consequently, implementers would benefit from the relative foci of such interventions being specified.

Further nuance in SEL categorization has important implications for implementation. The importance of implementation has been discussed in the field for some time (Durlak, 2016). However, concerns remain about the lack of empirical evidence regarding what intervention components ‘could be changed’ or ‘must not be’ changed. An ability to identify these elements is a critical step in advancing implementation science, as greater component specification can be addressed by offering a taxonomy to categorize elements of program provision, such as topic content and pedagogical approach. The classification of components has the potential to aid in issues of adaptation, as adaptation in response to context is an inevitable factor in program implementation and can lead to diminished outcomes (Taylor et al., 2017). Greater specification as to the content and approach foci for individual programs would equip potential local implementers with better knowledge with respect to choosing programs that target skills needed in their student bodies.

### Classifying SEL Programs Based on Their Core Components

Drawn from earlier work in the mental health field (e.g., Choroida & Daleiden, 2009) and targeted support (e.g., Sutherland et al., 2019), identifying core components offers a potential solution to the abovementioned issues. Defined as an “*irreducible unit of behavior change…that produces an observable, reliable result”* (Embry, 2004, p. 578), core components (also known as ‘kernels of practice’(Jones et al., 2017) or ‘common elements’ (Boustani et al., 2015)) are used to identify and classify specific elements of interventions. Thus, they present a potential opportunity to address the difficulties noted above. Studies have begun to identify granular program elements regarding the relative prevalence of the specific *practice* elements being imparted (e.g., social skills or emotional regulation). Notably, in an examination of 15 evidence-based programs selected from CASEL’s guide for elementary schools, Lawson et al. (2018) observed that there was an uneven distribution of practice elements within each CASEL core competency. For instance, although programs reported delivering content related to ‘self-awareness,’ there was a heavy emphasis on recognizing one’s own emotional states, with little comparative coverage of assessing one’s strengths and weaknesses. Otherwise unidentifiable through current research paradigms, this finding has implications for the specificity of effect and implementer’s fit for context, as discussed above.

Recent innovative work similarly demonstrates proof of concept by ‘dismantling’ several popular programs and identifying specific skills. Jones et al. (2017) distilled information from 25 SEL programs, including practice, instructional, and wider program features (e.g., family engagement). Notably, Jones et al. (ibid) did not examine cross-program similarities and differences. While this consideration was not the aim of this work, it is a gap regarding the broader implications or what constitutes ‘common’ SEL provision within evidence-based programs. In building on extant literature, a higher bar of evidence (drawing upon a smaller list of programs with a more extensive evidence base than those selected by Jones and colleagues) has been selected. Additional consideration has been applied as to whether categories, profiles, or taxonomies of provision both within and across programs are present within this selected sample.

### The Current Study

Opportunities for greater nuance in understanding SEL’s core components will benefit implementers and researchers. Providing better articulation of program specifics will offer a more nuanced categorization of SEL programming, with implications for fit for local context and refinements in future research when comparing program types.

Accordingly, the current study aims to examine content information of an identified short-list of evidence-based SEL programs to see whether a method of program categorization can be established.

## Method

The current study builds on previous work by drawing on the same dataset from Wigelsworth et al. (2022). This study examined core components regarding the inter-relations between practice elements *across* programs. The current study instead focuses on the prevalence of elements *within* programs, offering greater detail regarding available programs currently deployed in educational settings.

### Identification of Literature

As per Wigelsworth et al. (2022), a short-list for evidence-based programs was identified through a systematic literature review, utilizing the following sources:

- Contact with the authors’ networks and expert organizations (i.e., CASEL, European Network for Social and Emotional Competencies, individual researchers) requesting relevant sources.
- Systematic searches of bibliographic databases (i.e., EMBASE, ERIC, MEDLINE, and PsychINFO).
- A review of reviews—meta-analytic and systematic reviews were examined for relevant literature (e.g., Corcoran et al., 2018; Durlak et al., 2011; Sklad et al., 2012; Wigelsworth et al., 2016).
- Targeted searches of the following specific peer-reviewed journals were conducted: Prevention Science, Psychology in the Schools, Journal of Educational Psychology, Advances in School Mental Health Promotion, Learning and Individual Differences, School Mental Health, and Mental Health and Prevention.
- Searches of websites of relevant organizations (i.e., Early Intervention Foundation, Education Endowment Foundation, Child Trends, and The Wallace Foundation).
- Google Scholar was utilized to further identify any grey literature, as recommended by Haddaway et al. (2015).
- Subsequent reference harvesting from identified sources was also used.

Scoping was conducted for reviews published or made available between 31st December 1995^1^ and 1st June 2018. Thesaurus tools provided increased sensitivity of the searches; otherwise, the search strategy used a systematic combination of free-text words and phrases referring to an agreed keyword list. Key search terms (abstract and title) were: (social and emotional OR social OR emotion* OR well-being OR mental health) AND (program* OR promotion OR initiative OR pupil OR student* OR elementary* OR primary* OR school OR curriculum OR intervention).

Following Covid-19 disruptions, delaying the research preparation for publication, an updated search strategy was deployed to cover June 2018 (the end date of the initial search) to November 2022. This update ensured that no new programs had subsequently met the evidence threshold and should be included in the analysis. The updated strategy was as follows:

- A rerun of the original systematic search using PsychINFO (the database in which the original studies were found) with amended search dates (June 2018–November 2022). Any identified randomized trials of SEL interventions were collated and compared to the list of original programs identified (per the protocol specified below).
- A search of all program websites identified in the initial search. Evidence on each website (e.g., a summary of published studies) were compared to the criteria below to see whether they were eligible for inclusion (e.g., the publication of one or more randomized control trial since the end of the original search).

No new eligible programs were found as a result of the updated search strategy.

### Program Selection

As per Wigelsworth et al. (2022), programs eligible for inclusion met the following criteria: (a) targeted at least two SEL competencies, as defined by CASEL (see Table 1); (b) universally implemented (i.e., delivered to all pupils, irrespective of individual needs); (c) delivered during school hours on school premises; (d) delivered to elementary students (aged 4–12 years),^2^ and (e) has been evaluated in at least two randomized control trials (RCT), as identified through the process described above, producing a positive effect (e.g., showed significant findings consistent with an improvement in SEL based outcomes). Criterion (e) was identified first through collating studies from the previous review. From there, eligibility criteria (a)–(d) were assessed by examining program websites and materials. All information was obtainable through this approach. The purpose of criterion (e) was to set a benchmark for consistent positive evidence by narrowing the field of programs with a less robust success history. All studies had to be published within the past 20 years. Programs that had been evaluated in RCTs (Randomized Controlled Trials) but had not targeted the classroom component of the intervention (e.g., Incredible Years) or those evaluated in terms of SEL outcomes (e.g., self-management) but whose intervention content did not include SEL (e.g., Good Behavior Game), were excluded. In total, 13 programs were identified.

### Coding Process

For each identified program, the full curriculum and any accompanying materials were examined and coded. Consistent with the distillation approach (e.g., Chorpita et al., 2005; Lawson et al., 2018), individual instructions and activities from each program were examined and coded using a codebook (see supplementary materials) to classify individual practice elements. Codes were drawn directly from CASEL’s core competencies and specific skills, as defined in Table 1. This process aligns with practices from earlier reviews (e.g., Jones et al., 2017; Lawson et al., 2018).

‘Spiral’ curricula, where content is repeated across multiple years, were subject to further inspection. For programs with repetitive content where repetitive coding would not change the study results (i.e., identical prevalence of elements across years), a cross section of grades was chosen for coding (see Table 2). Otherwise, all grades were coded. Emergent codes were allowed to ensure the coding schedule accurately reflected the program content. However, all codes fit within the existing scheme.

**Table 2:** Program characteristics

Program	Country of origin	Number of sessions	Recommended delivery agent	Grades coded
FRIENDS	Australia	10	Teacher or external agent	Reception (Fun Friends) Grades 1–6 (Friends for Life)
I Can Problem Solve (ICPS)	USA	83 (R-3) 77 (Intermediate)	Class teacher	Reception- Grade 3 (R-3 version) Grades 3–6 (intermediate version)
INSIGHTS	USA	10	Class teacher	No differentiation of grades
KiVa Anti-bullying	Finland	20	Class teacher	Grades 3–5 (6–9 years version) Grades 5–6 (10–12 years version)
Positive Action (PA)	USA	140	Class teacher	Grade 6
Promoting Alternative Thinking Strategies (PATHS)	USA	52	Class teacher	Grade 1, Grade 4
Roots of Empathy (RoE)	USA	27	Trained instructor	No differentiation of grades
Second Step (SS)	USA	28	Class teacher	Grade 4
Social Skills Improvement System (SSIS)	USA	30	Class teacher	No differentiation of grades
Steps to Respect (StR)	USA	11	Class teacher	Grade 5
Tools for Getting Along (TFGA)	USA	20 (+ 6 booster)	Class teacher	R, Grades 1–6
Tools of the Mind (TotM)	USA	Practiced daily	Class teacher	Reception – Grade 2
Zippy’s Friends (ZF)	Ireland	24	Class teacher	No differentiation of grades

All coding was completed by three faculty members with at least 10 years in the field of psychological and educational intervention with expertise in social and emotional well-being and mental health and one PhD student with a specialty in developmental psychology and education. Consensus coding (Hill et al., 2005) was used to support the rigor of the coding process. Following trial coding, which enabled full agreement, simultaneous coding and cross-checking was employed, facilitating ad hoc discussion and decision-making for any ambiguous codes. Regular check-ins were used at an average of once per program chapter, where coders took turns explaining and justifying their coding choices.

Codes were summarized as part of each program’s content to ensure that larger programs with more activities were not over-represented in the final dataset. Summaries of aggregated codes were then presented descriptively according to the research question.

## Results

### Program Characteristics

As expected, programs were predominantly curriculum based, including a series of materials and activities for implementers to deliver in class. This content typically took the format of scripts for teachers to narrate and pupil participation through closed questioning (e.g., ‘how might someone feel if they couldn’t find friends to play with?’). Roots of Empathy was the least structured program, presenting a guide of activities to be delivered throughout the school year. Although all programs recommend a regular delivery schedule, there was a notable variability in the length of programs, with some offering as few as 10 sessions, and others offering more than 100 (Table 2).

### Prevalence of Core Competencies

Coding showed that program foci varied by core competency, with identifiable foci for all but one curriculum (Table 3). Program foci constituted any competency above 25% of the total program content. This identification was based on an assumed equal distribution of 20% per competency (whereby competencies would be evenly distributed across each domain), allowing for an additional 5% to accommodate a margin of error. There were unlikely to be an exactly equal number of activities per domain, given the practicalities of minor variations in lesson plans. Accordingly, perfectly even distribution was not necessarily expected, hence this accommodation. Table 3 shows that the short-list of evidenced-based programs represents CASEL’s core competencies. However, not all programs cover all of CASEL’s core competencies.

**Table 3:** Prevalence of CASEL’s core competencies by program

Program	Prevalence of CASEL core competency (% of program content)					Program foci
	Self-awareness (SA)	Self-management (SM)	Social awareness (SOC)	Relationship skills (RS)	Responsible decision making (RDM)	
FRIENDS	46	12.13	11.76	23.17	6.26	SA
I Can Problem Solve (ICPS)	8.21	3.86	44.45	9.66	33.82	SOC; RDM
INSIGHTS	14.52	0	15.53	0	68.4	RDM^*^
KiVa Anti-bullying	21.93	0	23.29	36.08	18.72	RS
Positive Action (PA)	45	13.34	19.59	5.84	16.25	SA
Promoting Alternative Thinking Strategies (PATHS)	36.13	10.71	10.38	14.03	27.74	SA;RDM
Roots of Empathy (RoE)	15.17	0	63.64	18.19	2.04	SOC^*^
Second Step (SS)	22.89	2.94	28.62	11.37	34.95	SOC;RDM
Social Skills Improvement System (SSIS)	14.58	15.24	17.5	41.01	11.67	RS
Steps to Respect (StR)	0	0	25.33	66.66	8.01	SOC;RS^*^
Tools for Getting Along (TFGA)	21.87	7.81	0	0	70.31	RDM^*^
Tools of the Mind (TotM)	7.41	67.60	9.26	15.74	0	SM^*^
Zippy’s Friends (ZF)	23.36	20.94	9.3	24.89	11.63	–

^*^Indicates more than ½ of program content dedicated to single competency

As Table 3 illustrates, no program showed an equivalent distribution of all five core competencies across program material, with several programs demonstrating more than 25% of program content was dedicated to either one or two core competencies. For five programs (INSIGHTS, RoE, STR, TfGA, and TotM), more than half of program content was dedicated to a single competency.

Tables 4, 5, and 6 offer further program content breakdown by individual core competency by noting the prevalence of specific skills (as defined in Table 1). This breakdown provides an overview of the breadth of each core competency addressed by programs and suggests a further nuance to program foci. For instance, although Zippy’s Friends covers the five core competencies, its focus on the specific skills is contained within each. Table 4 shows that although 23.6% of program content is dedicated to self-awareness, this is exclusively through identifying emotions, with no identifiable content addressing any other skill identified in CASEL’s framework: namely, accurate self-perception, recognizing strengths, self-confidence, or self-efficacy. Programs with dedicated foci demonstrate a similar pattern. For instance, 67.60% of the content for Tools of the Mind is dedicated to impulse control (25.93%) and self-discipline (41.67%), with no identifiable content targeting specific skills of stress management, self-motivation, goal setting, or organizational skills.

**Table 4:** Prevalence of core component by specific skill – Self-awareness and self-management

Program	% covered by the program										
	Self-awareness					Self-management					
	Identifying emotions	Accurate self-perception	Recognizing strengths	Self-confidence	Self-efficacy	Impulse control	Stress management	Self-discipline	Self-motivation	Goal setting	Organizational skills
FRIENDS	18.75	10.29	2.21	6.62	8.82	0.0	9.19	0.0	0.0	2.57	0.37
I Can Problem Solve (ICPS)	6.28	1.93	0	0	0	3.38	0	0.48	0	0	0
INSIGHTS	15.53	0	0	0	0	0	0	0	0	0	0
KiVa Anti-bullying	5.48	3.20	1.37	0.46	11.42	0	0	0	0	0	0
Positive Action (PA)	11.25	20	2.5	0.83	10.42	0.42	0	3.75	1.67	6.25	1.25
Promoting Alternative Thinking Strategies (PATHS)	25.41	8.73	0	1.66	0.33	4.97	2.10	0	0.55	3.09	0
Roots of Empathy (RoE)	1.52	7.58	4.55	0	1.52	0	0	0	0	0	0
Second Step (SS)	14.52	7.37	0	0	0	0	1.47	1.47	0	0	0
Social Skills Improvement System (SSIS)	3.08	5.83	0	5.67	0	6.32	2.92	6.00	0	0	0
Steps to Respect (StR)	0	0	0	0	0	0	0	0	0	0	0
Tools for Getting Along (TFGA)	12.50	1.56	7.81	0	0	0	7.81	0	0	0	0
Tools of the Mind (TotM)	4.63	2.78	0	0	0	25.93	0	41.67	0	0	0
Zippy’s Friends (ZF)	23.36	0	0	0	0	0	10.47	10.47	0	0	0

**Table 5:** Prevalence of core component by specific skill – Social awareness and relationship building

Program	% covered by the program							
	Social Skills				Relationship Skills			
	Perspective taking	Empathy	Appreciating diversity	Respect for others	Communication	Social engagement	Relationship building	Teamwork
FRIENDS	4.41	2.94	4.04	0.37	1.84	0.74	20.22	0.37
I Can Problem Solve (ICPS)	27.05	15.46	0.97	0.97	5.31	1.45	2.42	0.48
INSIGHTS	0	0	15.53	0	0	0	0	0
KiVa Anti-bullying	12.33	4.11	1.83	5.02	9.59	0.46	24.20	1.83
Positive Action (PA)	12.92	2.08	1.25	3.34	0.42	0.42	5.00	0
Promoting Alternative Thinking Strategies (PATHS)	3.20	2.54	1.10	3.54	6.19	0.66	5.30	1.88
Roots of Empathy (RoE)	7.58	42.42	0	13.64	16.67	0	1.52	0
Second Step (SS)	1.68	19.58	1.47	5.89	4.21	2.74	4.42	0
Social Skills Improvement System (SSIS)	0	8.75	2.92	5.83	14.59	20.58	2.92	2.92
Steps to Respect (StR)	1.33	0	0	24.00	4.00	17.33	45.33	0
Tools for Getting Along (TFGA)	0	0	0	0	0	0	0	0
Tools of the Mind (TotM)	5.56	3.70	0	0	0	0	9.26	6.48
Zippy’s Friends (ZF)	9.30	0	0	0	11.63	11.63	11.63	0

**Table 6:** Prevalence of core component by specific skill – responsible decision making

Program	% covered by the program					
	Responsible Decision Making					
	Identifying problems	Analyzing solutions	Solving problems	Evaluating	Reflecting	Ethical responsibility
FRIENDS	0	1.47	0.74	0.74	3.31	0
I Can Problem Solve (ICPS)	3.38	11.11	17.88	1.45	0	0
INSIGHTS	22.98	22.98	22.98	0	0	0
KiVa Anti-bullying	0	0	0	0	9.59	9.13
Positive Action (PA)	2.92	1.67	1.25	3.33	3.33	3.75
Promoting Alternative Thinking Strategies (PATHS)	5.75	2.54	9.61	3.54	2.54	3.76
Roots of Empathy (RoE)	0	0	2.04	0	0	0
Second Step (SS)	8.84	10.11	14.53	1.47	0	0
Social Skills Improvement System (SSIS)	2.92	0	0	0	0	8.75
Steps to Respect (StR)	0	2.67	2.67	2.67	0	0
Tools for Getting Along (TFGA)	0	18.75	50.00	1.56	0	0
Tools of the Mind (TotM)	0	0	0	0	0	0
Zippy’s Friends (ZF)	0	0	11.63	0	0	0

## Discussion

Through the distillation of core components, this study identified program foci, offering a more nuanced and differentiated representation of SEL provisions than currently available. Using distillation as a method, researchers identified the content of individual programs and examined the proportion of core competencies within each one. A categorization of program foci by which programs showed a particular emphasis on a subset of skills was then proposed. Thus, this process was demonstrated as a feasible method for providing more content information to support program categorization, which the researchers advocate.

Despite programs being commonly collated under the singular title of SEL and sharing an assumed common-evidence base (e.g., Jones et al., 2017; Corcoran et al., 2018; Cefai et al., 2018), almost all programs demonstrated specific foci regarding CASEL’s core competencies. Five programs showed significant foci, with over two-thirds of program content dedicated to specific skills (Table 3). For instance, almost 70% of INSIGHT's content was dedicated to skills relating to Responsible Decision Making, with zero content identified addressing Self-Management or Relationship Skills. Furthermore, INSIGHTS has a predominant focus on skills identified within the CASEL framework (see Table 1): notably identifying, analyzing, and solving problems (Table 6), with no content addressing, evaluating, or reflecting on identified problems or consideration of ethical responsibility. Similarly, Roots of Empathy addressed (unsurprisingly) empathy-based elements, with no content identified as addressing other social-skill elements, including appreciating diversity, social engagement, or teamwork. For programs covering all five core competencies (i.e., there was a % value above zero in each column shown in Table 3), no program showed a ‘balanced’ provision of SEL core competencies, allowing for a 5% margin of error. For instance, Zippy’s Friends, arguably the broadest of the examined programs (including content across all competencies), has twice as much content focused on self-awareness (23.26%) and self-management (20.94%) compared to social skills (9.3%) and responsible decision making (11.63%). Findings of relative foci are not directly related to program size or length. The longest program identified in the current research (Positive Action—140 lessons) has 45% of its content addressing self-awareness, with only 5.84% of program content addressing relationship skills. Again, when examining specific skills, less than 1% of program content addresses self-confidence or recognizing strengths, compared to the specific skill of self-awareness. PATHS (Promoting Alternative Thinking Strategies), with 52 separate lessons, has approximately three times more of its content dedicated to imparting self-awareness skills (36.13%) than self-management (10.71%) or social skills (10.38%). In summary, despite the implied breadth of SEL, based on analysis of the short list of evidence-based programs within the current study, it would be difficult to select a program that satisfactorily encompasses the full gamut of competencies and skills contained within CASEL’s definition (Table 1). Thus, these findings provide a strong case for identifying program foci to better understand the comparative emphases of skills for individual programs.

### Implications of the Research

This paper does not argue that programmatic content is necessarily limited or restricted by focusing on specific core competencies, specific skills, or both. Indeed, there is a strong call to ensure that programs fit the need of specific contexts when preparing to adopt SEL (Oberle et al., 2016). Although there is some broad delineation in current programs to help identify the intended purpose or implied focus of core competencies they contain (e.g., KiVA and Steps to Respect are signaled as specifically addressing bullying), potential implementers are arguably not well resourced to identify specific SEL program content without further specification, such as that demonstrated in the current study. Although the current research may offer some immediate benefits to practice regarding a more nuanced understanding of the short-list of programs contained in the current list, this represents a very small portion of available program content. The methods used to identify foci were arguably resource intensive and, therefore, currently offer limited benefit to practitioners wishing to code programs not included within current evidence. Accordingly, research implications are mainly directed toward program developers and researchers in the field.

Current program descriptors (such as ‘what works’ evidence repositories, as discussed in the introduction) do not provide sufficient detail for more nuanced choices. Fit to context is associated with successful outcomes (Edwards et al., 2005; Meyers et al., 2015), and full manuals or summaries of programmatic content (such as that demonstrated in the current study) are not available to potential implementers enabling an informed choice. Therefore, an opportunity emerges to support the successful adoption of SEL programming through more nuanced content descriptions as part of the summary information provided by programs.

This provision may take the form of a short ‘ingredients’ list (imaginatively, perhaps not dissimilar to those displayed on food packaging, indicating content proportions). There is already some tentative move toward demonstrating the potential efficacy of such an approach. For example, Jones et al. (2017) demonstrate a similar distillation of program content in their examination of wide range of school-based programming, with a similar demonstration of ‘ingredients’ or ‘kernels.’ While Jones and colleagues do not identify a specific foci taxonomy, this could be achieved. For instance, using the categories presented in the current study. However, the adoption of foci-based taxonomy through content distillation is not necessarily straightforward, as CASEL’s framework is not the only model for examining the wide constellation of social, emotional, and personal skills program content contains. This issue is discussed further below.

A further implication regarding the current program landscape is the extent to which skills hypothesized as being integral to SEL provision are otherwise absent across the identified programs. For instance, self-motivation is a specific skill cited within the constellation of competencies and abilities of Social and Emotional Learning. However, in the 13 examined short-listed programs, only two programs address this content (Positive Action and PATHS). Moreover, within these programs, these skills represent less than 2% of the program content. Lack of skill coverage is also true for ‘organizational skills,’ contained within CASEL’s core competency of self-management. Only two programs were identified as including content addressing this specific skill (FRIENDS and Positive Action) and, again, representing less than 2% of the program content. There is a question as to whether the current evidence base of SEL programming sufficiently covers the full range of skills associated with the *hypothesized* model of SEL. Perhaps programs with foci in otherwise undeveloped areas (e.g., self-management) have yet to accrue a sufficient evidence base to be reflected in the current short-list of programs, as per the current study’s threshold for demonstrating the evidence of success. Extant work in the field would potentially support such a hypothesis. For instance, Jones and colleagues’ (2017) review of 26 programs shows some interventions focusing heavily on self-management. Additionally, the program ‘RULER’ (Brackett et al., 2019) showed that 51% of the program content was dedicated to emotional/ behavioral regulation. Notably, Jones’s review used a lower threshold for evidence inclusion than the current research. Therefore, it is possible that self-management *is* part of a program’s offering regarding SEL programming but, in effect, has a weaker evidence base. Although possible, this may be unlikely, as these skills are seen as malleable given the evidence of successful intervention in the context of education in other arenas (e.g., Pandey et al., 2018).

Another alternative explanation for the results is that the distillation process does not sufficiently capture the more subtle or underlying skill-building that may be inherent within a program. ‘Sustained commitment to a task’ is an example of a behavioral regulation strategy. However, this example would not necessarily need to be included as an explicit instruction with SEL materials, meaning it would not be captured using the current approach. Therefore, self-management skills may be more ubiquitous within SEL programs than results suggest. This hypothesis aligns with absent areas identified in Jones and colleagues’ report, as the absence of content is also noted for similar skills. For instance, in their analysis, an average program content of 10% addressed attentional control, and 15% examined emotional and behavioral regulation, with some individual programs offering no zero content in this regard (e.g., Positive Action and Playworks, respectively). A further explanation is that for both Jones et al. and the current study, programs with a focus on self-management skills are identifiable though alternative frameworks. Search terms for both studies required identifying social terms, emotional terms, or both, which precludes more focused frameworks. For instance, Boekaerts’ dual-processing self-regulation model (Boekaerts & Corno, 2005) discusses how self-learning goals interact with well-being. However, by virtue of a focus on academic goals as a *proximal* or immediate outcome of self-regulation (where in SEL models, attainment is a distal outcome; e.g., Greenberg & Jennings, 2009), intervention approaches focusing on self-regulation as an explicit and immediate academic skill would not be classified as SEL, despite sharing similar underlying program theory.

The lack of more precise developmental linkages in CASEL’s current framework has implications for theorists regarding how SEL continues to be conceptualized (e.g., is self-management an explicitly ‘taught’ skill?). Implications arise for implementors for identifying and selecting programs regarding their intended outcomes (e.g., is SEL optimal for imparting self-regulation skills, or should other programs be considered?) and for program developers regarding content coverage (e.g., should there be more focus on self-management and regulation content within SEL programming?). This lack of a consensus about the precise constructs contained with SEL (Schoon, 2021) is an issue, and until there is further clarity, such questions will be difficult to answer.

As a brief and final point, there is an implication for research regarding the potential use of identified program foci as an attempt to address long-standing issues with prominent levels of heterogeneity that have plagued meta-analytic studies in this area (e.g., Corcoran et al., 2018; Durlak et al., 2011; Sklad, et al, 2012; Wigelsworth et al., 2016). All the short-listed programs within the current study have featured as part of meta-analyses examining the overall impact of SEL. Most meta-analyses examining SEL have summarized program outcomes over several distinct categories (e.g., attitudes toward self, pro-social behavior, and emotional competence). However, they have made no accommodation for program foci, accepting SEL as a singular intervention unit. Utilizing a classification system, such as the program foci identified in Table 3, as a mediator to examine whether program foci match intended outcomes should help address this issue of heterogeneity.

### Limitations

An important caveat to the findings of the current study is the lack of consideration of the wider context and structure of SEL implementation. Core content components are only one piece of the puzzle for successful SEL implementation. Consequently, there are further opportunities to consider other key elements in delivery; for instance, pedagogical approaches (e.g., how key content is delivered) and broader approaches, such as changes to school-wide climate and culture (e.g., school-wide activity and events, such as assemblies, alongside professional development and training). Such approaches will have their own challenges, requiring different coding frameworks. Thus, they are beyond the scope of this paper.

A further consideration is selecting CASEL’s core competencies and specific skills as the study’s frame of focus. As briefly discussed above, there are alternative frameworks for considering SEL. In this respect, other elements of program content may have been forfeited. For instance, mindfulness and character are related to wider definitions of SEL (Schoon, 2021). However, the current study did not use CASEL’s core components specifically in identifying relevant literature (using the broadest terms of ‘social and/or emotional’). However, 9 of the 13 programs identified within the current study feature in CASEL’s program guide as recommended SEL programs, and the remaining 4 have been independently identified as SEL programs that could be considered potentially eligible for CASEL’s program guide (e.g.Espelage, 2015; Kozina, 2021; Schonert-Reichl et al., 2012). Findings suggests that the study did capture programs well-suited to content analysis using CASEL’s core competencies as a framework.

## Conclusion

The current study findings indicate a revised and more nuanced picture of SEL provision than what is currently available through a process of identifying core components and subsequent classification of program foci. In turn, a suggestion is generated that there may be key gaps in the provision of CASEL’s full spectrum of core competencies when considering only those programs with a robust history of success. Given the global ubiquity of SEL programming, there is increasing consideration of the importance of context and fit for SEL programming (Oberle et al., 2016). Therefore, this is timely in supporting the contextualization and incorporation of more informed choices in SEL provision. The researchers submit that the identification of core components could support the development and successful implementation of SEL and have set out a (cautious) approach through which this might be achieved in future work.

## Footnotes

1 The start date for the literature search coincides with the publication of Daniel Goleman’s “Emotional Intelligence,” broadly attributed as a catalyst for the rise in popularity of SEL-based approaches in school (Elias & Weissberg, 2009).

2 Elementary school represents the earliest time at which all school children receive mandatory education in a systematic and universal manner (i.e., within schools and classrooms) (McClelland et al., 2017), and therefore the majority of SEL programming is targeted at this level.
